# Expectation Suppression in Early Visual Cortex Depends on Task Set

**DOI:** 10.1371/journal.pone.0131172

**Published:** 2015-06-22

**Authors:** Elexa St. John-Saaltink, Christian Utzerath, Peter Kok, Hakwan C. Lau, Floris P. de Lange

**Affiliations:** 1 Donders Institute for Brain, Cognition and Behaviour, Radboud University, Nijmegen, the Netherlands; 2 Department of Psychology, University of California Los Angeles, Los Angeles, California, United States of America; Centre de Neuroscience Cognitive, FRANCE

## Abstract

Stimulus expectation can modulate neural responses in early sensory cortical regions, with expected stimuli often leading to a reduced neural response. However, it is unclear whether this expectation suppression is an automatic phenomenon or is instead dependent on the type of task a subject is engaged in. To investigate this, human subjects were presented with visual grating stimuli in the periphery that were either predictable or non-predictable while they performed three tasks that differently engaged cognitive resources. In two of the tasks, the predictable stimulus was task-irrelevant and spatial attention was engaged at fixation, with a high load on either perceptual or working memory resources. In the third task, the predictable stimulus was task-relevant, and therefore spatially attended. We observed that expectation suppression is dependent on the cognitive resources engaged by a subjects’ current task. When the grating was task-irrelevant, expectation suppression for predictable items was visible in retinotopically specific areas of early visual cortex (V1-V3) during the perceptual task, but it was abolished when working memory was loaded. When the grating was task-relevant and spatially attended, there was no significant effect of expectation in early visual cortex. These results suggest that expectation suppression is not an automatic phenomenon, but dependent on attentional state and type of available cognitive resources.

## Introduction

Stimulus expectation can modulate neural responses in early sensory cortical regions, with expected stimuli often leading to a reduced neural response [1, 2]. This effect has been found in visual [1, 3, 4] and auditory [5, 6] cortices, and in both electrophysiological [1, 5] and haemodynamic [3, 4, 7] measurements.

Is expectation suppression an automatic process that happens outside the focus of attention? Several studies suggest that this is the case. A reduced neural response for predictable stimuli has been found during passive viewing [8], as well as when stimuli are fully task irrelevant [7], supporting the idea that suppression occurs automatically, whenever sensory input is predictable. In contrast to this notion however, other authors found no effect of expectation on sensory activity when stimuli were unattended [9], suggesting that expected background stimuli are not automatically suppressed.

One potential explanation for these conflicting results could be that the specific task set a subject is engaged in, and thereby the available resources, may determine whether stimulus expectations modulate the sensory response. For example, load theory [10] states that the processing of task-irrelevant stimuli is determined by the type and level of resource load posed by a given task. In line with this, previous research has shown that task set can have a profound effect on the extent to which items in the visual background are processed [11, 12]: irrelevant background stimuli tend to be suppressed during tasks that load perceptual resources, whereas there is no suppression for background stimuli when working memory is taxed. Therefore, expectation suppression of background stimuli might be especially pronounced during tasks with a high perceptual (but not working memory) load. Critically, in support of this idea, the study which found that predictable background stimuli were not suppressed by expectation used a paradigm that loaded working memory resources [9].

Therefore, in the current study we asked whether expectation suppression for background stimuli depends on the type of available resources. To investigate this, we compared the neural response to predictable and non-predictable visual background stimuli during tasks that placed a higher load on either perceptual or working memory resources. If task set interacts with the expectation effect, this would indicate that expectation suppression is dependent on how processing resources are engaged by the task at hand. Conversely, if no such interaction is present, this would indicate that expectation suppression is independent of the type of available resources. To compare the effect of spatial attention, we included a task that made the background stimulus task-relevant. Due to previous reports of expectation suppression for task-relevant, spatially-attended stimuli, we hypothesized that predictable stimuli would be suppressed relative to non-predictable stimuli during this task. With this design we aimed to elucidate the conditions for which sensory input is suppressed by expectation.

## Materials and Methods

### Participants

Thirty-five healthy right-handed individuals (25 females, age 22 ± 4, mean ± standard deviation (SD)) with normal or corrected-to-normal vision gave written informed consent to participate in this study. Experimental procedures were approved by the local ethics committee (Commissie Mensgebonden Onderzoek region Arnhem-Nijmegen, the Netherlands). Data from two subjects were excluded due to chance level performance on one or more of the tasks.

### Stimuli

**Fig 1:**
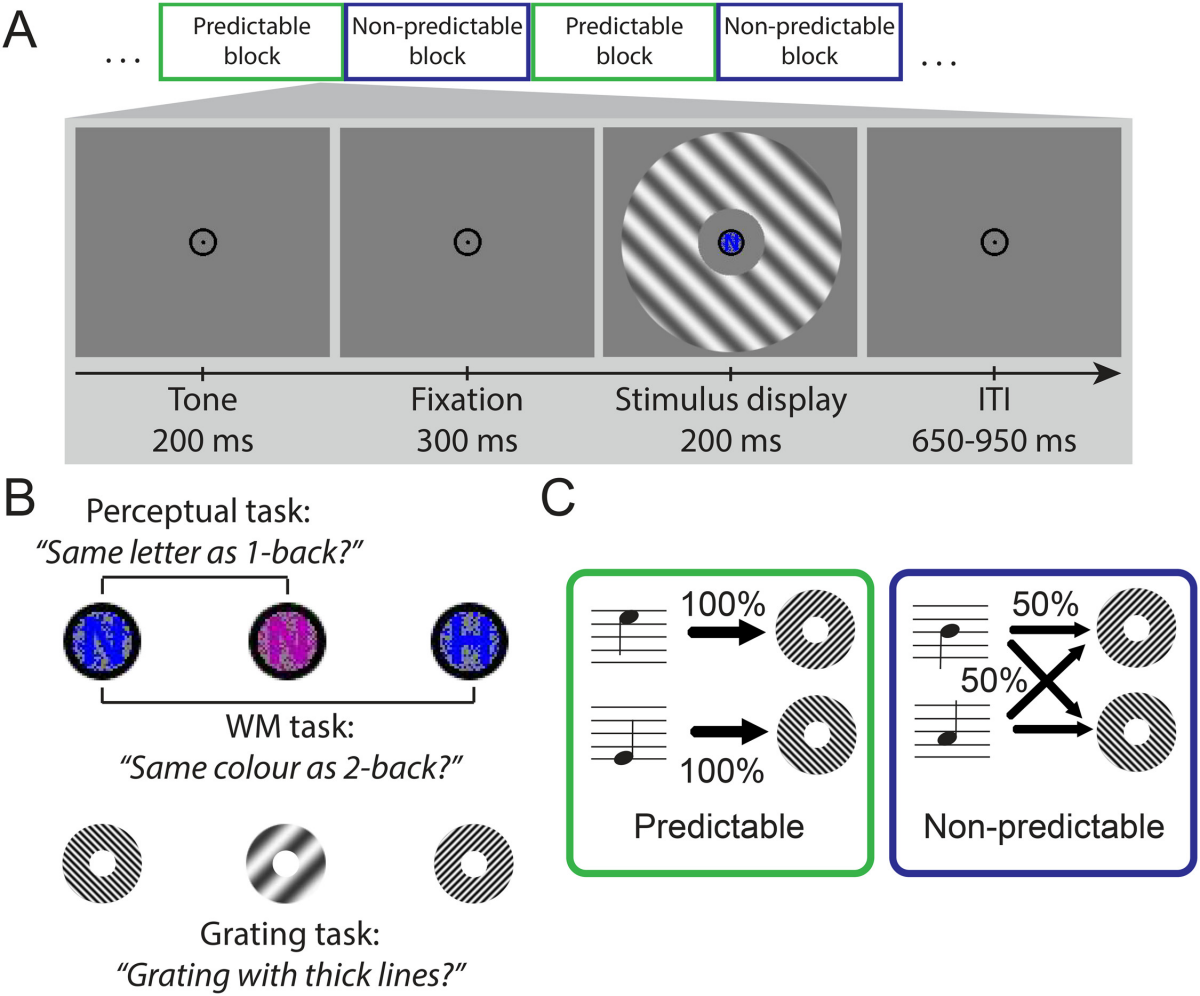
Experimental paradigm. (A) During each of the three tasks, stimuli were presented in predictable and non-predictable blocks, alternating every 12 trials. Each trial started with an auditory tone that either perfectly predicted the orientation of the subsequent grating stimulus (45° or 135°), or carried no orientation information. (B) Participants performed two tasks on the stimuli in the fixation bull’s-eye. During the perceptual task, targets were 1-back letter repetitions (the magenta ‘N’ is a target) that were difficult to perceive due to added noise. During the working memory task, targets were 2-back colour repetitions (the blue ‘H’ is a target) that were easy to perceive (as the whole inner ring has the same colour) but taxed the working memory system more strongly than the 1-back task. During the grating task, participants responded to the spatial-frequency of the grating stimuli. Targets had lower spatial-frequency than non-targets. (C) Pairing between auditory tones and grating orientations. In predictable blocks, tones predicted grating orientation with 100% accuracy. In non-predictable blocks, the tones provided no orientation information. ITI = inter-trial interval.

Stimuli were generated using MATLAB (MathWorks, Natick, MA, US) in conjunction with Psychophysics Toolbox [13]. In the behavioural session, stimuli were displayed on a Samsung SynchMaster 940BF monitor (60 Hz refresh rate, 1280 X 1024 resolution). In the fMRI session, stimuli were displayed on a rear projection screen using a luminance-calibrated EIKI projector (60 Hz refresh rate, 1024 X 768 resolution) which participants viewed through a mirror. A fixation “bull’s-eye” (outer ring 0.8° of visual angle) was presented at the centre of a gray background throughout each task. On each trial, a grating annulus (outer diameter: 15° of visual angle; inner diameter: 2°) of luminance-defined sinusoids at 80% contrast was displayed around the fixation bull’s-eye (200 ms; Fig 1A). Gratings were oriented at either 45° or 135°, with a phase randomly selected from 10 possibilities, evenly spaced between pi and 2pi. To mitigate afterimages, the phase of the grating was inverted halfway through stimulus presentation. On each trial, gratings had one of two possible spatial frequencies (mean: 1.5 cpd), with the specific spatial frequency values set for each individual by a staircasing procedure (see below). Simultaneously with the grating presentation, coloured letters were presented in the centre of the fixation bull’s-eye, with noise of the same colour superimposed (Fig 1B). Six letters (A, H, N, R, T, Z) and six colours (red, blue, green, cyan, yellow, magenta) were used. The number of coloured pixels that degraded the letters was similarly set by a staircasing procedure. The titration of these stimulus parameters for each subject ensured that tasks were matched on difficulty. Auditory cues that were played before each stimulus consisted of four pure tones (329, 440, 493 and 659 Hz) that were played for 200 ms.

### Experimental Design

To maximize sensitivity to the effects of predictability, stimuli were presented in a block design. On each trial, an auditory tone was presented 500 ms before the onset of the visual stimulus display. These auditory tones contained predictive information about upcoming visual stimuli, similar to previous studies [3, 7, 14–16]. During predictable blocks, the auditory tones cued the orientation of the upcoming grating with 100% validity. During non-predictable blocks, the auditory tones provided no orientation information (50% validity; Fig 1C). Then, the stimulus display was presented, which consisted of both the grating annulus and the degraded coloured letter at fixation. The stimulus display remained on screen for 200 ms (see Fig 1A), followed by an inter-trial interval jittered between 650 and 950 ms. We reasoned that including this jitter between trials would increase the pairing between tones and gratings *within* trials(which had a fixed temporal distance of 500 ms), relative to stimuli *across* trials. Hereby we aimed to increase the salience of the predictable statistical structure of the tone-orientation pairings. Cue validity alternated every 12 trials (18 s), with one trial presented on average every 1.5 s (range 1.35–1.65 s). Using cross-modal cues to manipulate conditional probabilities has advantages over inducing expectations by manipulating base rate occurrence of stimuli, since the latter could cause differences in stimulus-specific adaptation [17, 18].

We manipulated task set while diverting spatial attention away from the grating stimuli by loading either perceptual resources or working memory resources at the fovea (see Fig 1B). Following the study of Yi et al. [12], we induced a high working memory load by a 2-back task on easy to perceive stimuli, whereas a high perceptual resource load was induced by a 1-back task on visually degraded stimuli. Instead of manipulating the level of resource load, we elected to use only high load conditions with carefully titrated stimuli to match performance, so as to facilitate a direct comparison between the different types of load (rather than the amount of load). During the perceptual task, participants responded to the degraded letters within the fixation bull’s-eye: a letter was a target if it was the same as during the previous stimulus display (1-back task). This task was perceptually challenging because the letters were occluded by noise of the same colour, making them difficult to identify. During the working memory task, participants responded to the colour of the letters and noise pixels within the fixation bull’s-eye: a colour was a target if it was the same as the stimulus display presented two trials ago (2-back task). The 2-back task required participants to encode, maintain and compare more items in working memory than the 1-back task, and thereby it placed a higher load on working memory. We also included a task in which attention was directed to the grating. During this task, participants responded to the spatial-frequency of the grating stimuli: low-spatial frequency gratings were targets. Therefore, the predictable grating stimuli could be either unattended with a load on perceptual resources (letter task); unattended with a load on working memory resources (colour task); or attended and task-relevant (grating task). For all tasks, participants were instructed to respond after every trial by indicating ‘target’ (button 1) or non-target (button 2). Each task had the same proportion of targets (33%), and participants were instructed to maintain fixation during all tasks.

Per scanner run of 5.4 min, participants performed 14 blocks of one of the tasks, with four fixation blocks interspersed. During fixation blocks, only the bull’s eye was presented and participants were instructed to maintain fixation. Stimulus sequences were repeated for three runs such that each task was performed on the same stimuli. Participants cycled through the tasks until each had been performed three times, yielding 504 trials per task. Task order was counterbalanced across participants. Feedback (target accuracy (%), number of timeouts to targets, non-target accuracy (%), and number of timeouts to non-targets) was displayed for 2 s at the end of every run.

After the main experiment, we carried out two additional scans: one to identify voxels that were maximally responsive to the grating stimulus and one to retinotopically delineate early visual cortices. During the grating localizer, full contrast gratings of the same size and position as the main experiment were presented. Gratings were flickered at 2 Hz for 14.4 s at eight orientations (22.5, 45.0, 67.5, 90.0, 112.5, 135.0, and 157.5 degrees). Each orientation was presented four times in a pseudo-random order. To ensure fixation, participants’ task was to detect two letters (‘X’, ‘Z’) in a stream of letters within the fixation bull’s-eye. During the retinotopy scan, a 90 degree wedge stimulus consisting of a flashing black-and-white checkerboard pattern (3 Hz) rotated in 30 degree steps (1 position per TR) on a black background. Nine cycles of clockwise and counter clockwise rotation were presented. Participants’ task was to detect unpredictable changes in the colour of the central fixation point (white to black), which occurred four to eight times per 36 s block. During both scans, participants responded to target events with a button press.

### Behavioural Session

To familiarize participants with the tasks and to ensure all tasks were equally difficult, stimuli were calibrated prior to the scanning session. Participants were explicitly taught the relationships between the informative tones and the grating orientations, and their knowledge of these relationships was explicitly tested at the beginning and the end of the training session as well as directly preceding the fMRI session. Participants were trained on the working memory task until they could achieve better than 60% target accuracy on one run of this task (using identical presentation parameters to the fMRI session). Participants who could not achieve this performance were excluded from further participation. The stimuli from the perceptual and grating tasks were adjusted using an adaptive staircase-procedure [19] set to the accuracy that the participant attained on the working memory task. First, accuracy on the perceptual task was manipulated by adjusting the number of coloured noise pixels surrounding the letter. Then, accuracy on the grating task was manipulated by adjusting the difference between high and low spatial-frequency gratings (around a mean of 1.5 cpd). During both tasks, stimulus timing was identical to during the fMRI experiment, except that participants had the opportunity to pause every four blocks while stimulus parameters were updated according to the staircase value. This continued until the target accuracy was reached. This procedure matched performance on the different tasks within participants, while allowing differences in overall performance between participants. The behavioural session was held within seven days prior to the scanning session. Just before entering the scanner, participants were exposed to the relationship between the informative tones and the grating orientations with 24 practice trials.

To determine whether expectation or task had an effect on behaviour during the fMRI session, we performed a repeated measures ANOVA (rmANOVA) with factors expectation (predictable; non-predictable) and task (perceptual; WM; grating) on accuracy and RT. A main effect of task on RT was further investigated using post-hoc t-tests. Finally, to probe whether expectation had an effect when the predictable stimulus was attended and task-relevant, we compared accuracy and RT during predictable versus non-predictable blocks for the grating task using post-hoc t-tests.

### fMRI Acquisition and Analysis

Functional images were acquired using a 3T Trio MRI system (Siemens, Erlangen, Germany) using a 32-channel head coil, with a 3D EPI sequence (TR 1.8 s, 64 transversal slices, 2 x 2 x 2 mm in-plane resolution, TE 25 ms, field of view 224 mm x 224 mm, GRAPPA acceleration factor of 2). A high resolution anatomical image was collected using a T1-weighted MP-RAGE sequence (TR 2.3 s, TE 3.03 ms, 1 x 1 x 1 mm in-plane resolution, GRAPPA acceleration factor of 2).

Data were pre-processed using SPM8 (http://www.fil.ion.ucl.ac.uk/spm; Wellcome Trust Centre for Neuroimaging, London, UK). The first four volumes of each task run were discarded to allow for time to achieve initial equilibrium. Functional images from all sessions were spatially realigned to the mean image, and the resulting movement parameters, their first order derivatives and the square of these derivatives were included as nuisance regressors in the general linear model (GLM). The structural image was coregistered to the functional volumes.

Functional data from each subject were modelled using a block design approach, within the context of the General Linear Model (GLM) that included the data from all nine task runs. A 128 s high-pass filter removed low-frequency oscillations. Regressors for the task conditions were specified per run (180 scans), and convolved with SPM8’s canonical hemodynamic response function, which is comprised of the sum of two gamma functions. In addition to task regressors (predictable, non-predictable and fixation), the motion parameters as described above and a regressor to capture adaptation effects were included. The resulting beta-weights for predictable, non-predictable, and fixation for each run were analyzed in Matlab using in-house code. For each run, the fixation beta weight was used to normalize the beta-weights for predictable and non-predictable, and then the normalized betas were averaged across the three runs of each task. A separate design matrix was constructed for the grating localizer data, with regressors for stimulation, fixation, and the motion parameters (with derivatives as discussed above).

Freesurfer (surfer.nmr.mgh.harvard.edu/) was used to inflate the cortical surface of each participant’s T1-weighted structural image and to analyze the functional data from the retinotopy session. Polar-angle maps were generated using Fourier-based methods and projected onto the surface of the inflated cortex according to established methods [20], allowing retinotopic areas within early visual cortex to be visually identified and delineated. Freesurfer and SPM functions were used to convert the retinotopic labels from surface to volume space and to transform them into regions of interest (ROIs).

Within these retinotopic ROIs of V1-V3, we averaged the task-related activity of the 150 voxels that were most responsive to the grating stimulus during the localizer. To test whether the effect of expectation was influenced by task demands, we performed an rmANOVA with factors expectation (predictable; non-predictable) and task (perceptual; WM; grating) on the normalized beta weights averaged per task. This rmANOVA was performed separately for each ROI. A main effect of task and an interaction between task and expectation were found. Because we wanted to investigate whether expectation suppression for background stimuli depends on the type of available resources, we compared the two fixation tasks directly, using an rmANOVA with factors expectation (predictable; non-predictable) and task (perceptual; WM) on the normalized beta weights averaged per task. To verify that the expectation effect also differed between the perceptual and grating tasks, we compared the expectation effects during these tasks with an rmANOVA with factors expectation (predictable; non-predictable) and task (perceptual; grating).

To further examine the interactions, we performed post-hoc t-tests on the expectation effects for each task separately, within each ROI. These expectation effects were calculated by subtracting predictable from non-predictable beta weights, such that positive values would indicate a lower response to predictable than non-predictable gratings (expectation suppression). We statistically evaluated the robustness of the effect using 150 voxels. Additionally, to assess whether the effect was stable across different voxel selection criteria, we calculated the expectation effect and the corresponding standard error of the mean (SEM) over a range of included voxels (50 to 300 voxels, in steps of 50 voxels).

Additionally, to investigate neural activity modulations in areas outside early visual cortex, we performed a separate, whole-brain analysis. Preprocessing followed a similar pipeline, except that subjects’ T1 scans were normalized to MNI space. Functional images were brought into MNI space using the anatomical normalization parameters, and then smoothed with 8 mm kernel. The same GLM outlined above for the non-normalized images was applied to these data at the subject-level. The resulting beta weights were taken to a second (between-subjects) level, and tested using one-sample t-tests over linear combinations of the beta weights across subjects. To specifically test for areas showing the same resource-dependence of expectation suppression as found in the early visual cortex during the tasks at fixation, we specified a contrast to evaluate where there was greater expectation suppression during the perceptual task than the working memory task. Statistical inference was performed at the group-level using a cluster-level statistical test to assess clusters of significant activation [21]. We used a familywise error (FWE) corrected cluster threshold of *p* < .05, with the spatial extent of clusters defined by a voxel threshold of *p* < .001 at the whole-brain level.

## Results

### Expectation suppression for unattended stimuli

#### Behaviour

Participants performed equally well on all three tasks (accuracy 85.6%, 87.8%, and 87.9% for perceptual, WM, and grating tasks, respectively, rmANOVA over all tasks: *F*_2,64_ = 1.34, *p* = 0.27), demonstrating that participants followed task instructions and task difficulty was matched. There was no influence of grating predictability on either accuracy (rmANOVA over all tasks: *F*_1,32_ = 2.48, *p* = 0.13) or RT (rmANOVA over all tasks: *F*_1,32_ = 2.13, *p* = 0.15). There was a significant effect of task on RT (rmANOVA over all tasks: *F*_2,64_ = 23.45, p<0.001). Due to the additional time necessary for evidence accumulation of perceptually challenging stimuli, responses during the perceptual task (mean RT: 564 ms) were slower than during the grating task (mean RT: 524ms; *t*_32_ = -4.21 *p* = 0.002), and grating task responses were slower than during the working memory task (mean RT: 504 ms, *t*_32_ = 2.22, *p* = 0.035). While valid expectations about task-relevant stimulus features often lead to behavioural improvements, post-hoc t-tests on behavioural data from the grating task confirmed that there was no behavioural benefit of predictability on accuracy (*t*_32_ = -1.03, *p* = 0.31) or RT (*t*_32_ = -0.20, *p* = 0.84). For the grating task, expectations about stimulus orientation are not directly relevant to the task at hand (a spatial-frequency task) and therefore may not provide a behavioral benefit.

#### Visual cortex activity

**Fig 2:**
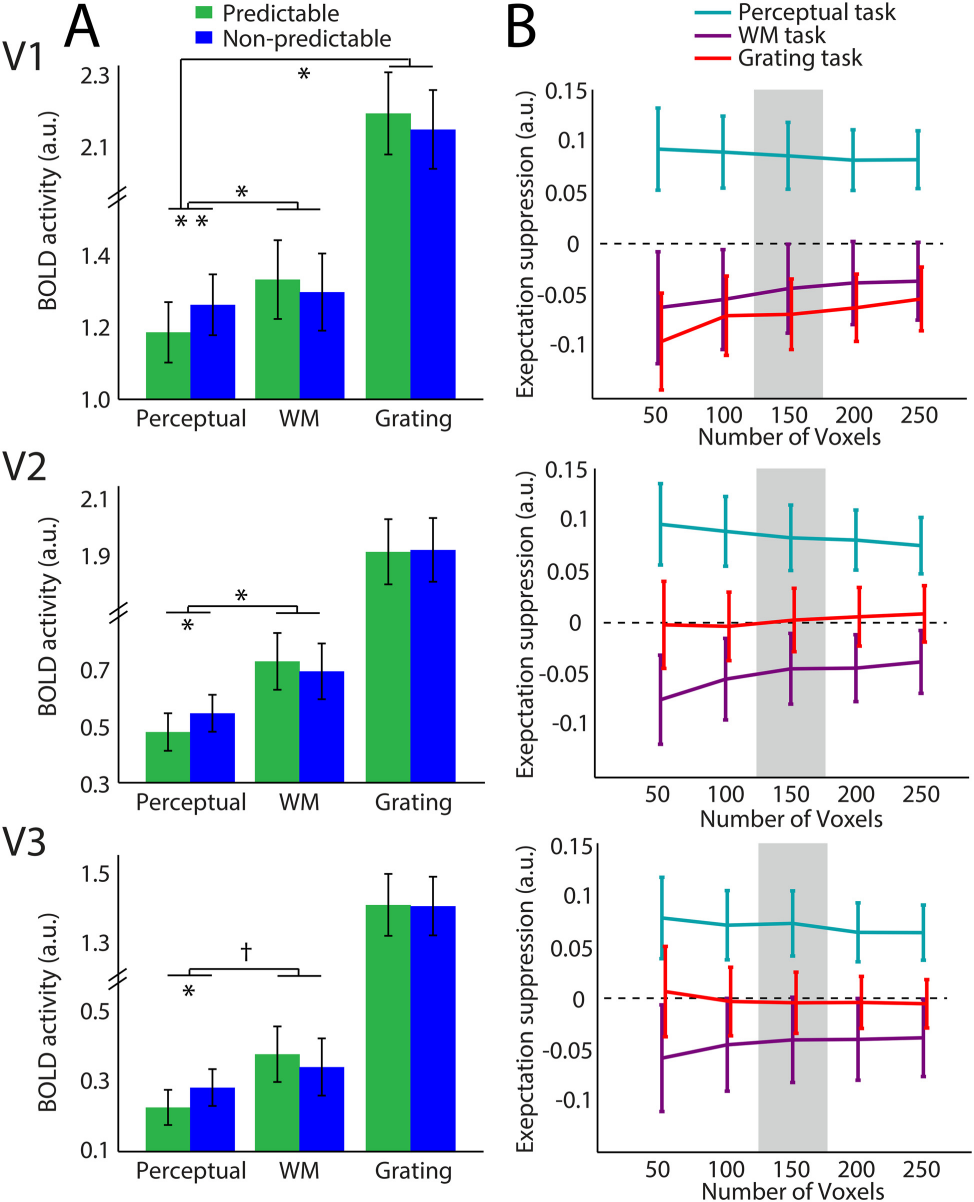
Expectation suppression for unattended stimuli depends on task set. (A) Amplitude of BOLD response (in arbitrary units; “a.u.”) within the 150 most grating-responsive voxels for predictable (green) and non-predictable (blue) stimuli, per visual area, per task. In each area, there was a reduced neural response to predictable stimuli within the perceptual task, but not during the working memory task or the grating task. Error bars reflect unbiased within-subjects corrected SEM [22, 23]. (B) Expectation effects per number of included voxels, defined as the neural response to non-predictable minus the response to predictable gratings. Positive values indicate a lower response to predictable than non-predictable gratings (expectation suppression). Values close to zero indicate that expectation does not have an effect. Error bars reflect SEM. Grey region indicates the number of voxels depicted in A. Significance reported (if any) on the basis of t-tests for effect ≠0. **p < .01; *p <05; Ɨ 0.1> *p* >.05

To probe whether the effect of expectation depended on the task participants engaged in, we investigated the BOLD response in voxels in primary visual cortex that responded to the grating stimuli (see Methods). An interaction between expectation suppression and task would indicate that the effect of expectation is influenced by participants’ task set. This is indeed what we found: the effect of expectation depended on task (rmANOVA over all tasks, V1: *F*_2,64_ = 4.15, *p* = 0.020; V2: *F*_2,64_ = 3.44, *p* = 0.038). We were particularly interested to know whether the expectation effect differed between the two tasks in which the predictable stimulus was task-irrelevant, since these tasks differed only in the relative load they placed on perceptual and working memory resources, but were matched on the locus of spatial-attention and the task-irrelevance of the grating. Indeed, the expectation effect depended on how processing resources were constrained by task demands: there was greater expectation suppression during the perceptual task than during the working memory task (rmANOVA over fixation tasks, V1: *F*_1,32_ = 5.08, *p* = 0.0312; V2: *F*_1,32_ = 5.95, *p* = 0.020; Fig 2A). Expectation suppression was also greater during the perceptual task than during the grating task in V1 (rmANOVA over perceptual and grating tasks, V1: *F*_1,32_ = 7.02, *p* = 0.012; V2: rmANOVA, *F*_1,32_ = 2.03, *p* = 0.17). In fact, there was a significantly reduced neural response to predictable stimuli only during the perceptual task (V1: *t*_32_ = 2.90, *p* = 0.0068; V2: *t*_32_ = 2.54, *p* = 0.016), but not during the working memory task (V1: *t*_32_ = -0.94, *p* = 0.35; V2: *t*_32_ = -1.17, *p* = 0.25) or the grating task (*t*_32_ = -1.52, *p* = 0.14; V2: *t*_32_ = 0.26, *p* = 0.79).

V3 displayed the same pattern of results, but the interaction between task and expectation did not reach significance across any of the task combinations (rmANOVA over all tasks: *F*_2,64_ = 2.80, *p* = 0.068; rmANOVA over fixation tasks: V3: *F*_1,32_ = 3.90, *p* = 0.057; rmANOVA over perceptual and grating tasks, *F*_1,32_ = 2.40, *p* = 0.13).

These results were obtained on the basis of the 150 most grating responsive voxels (as determined by an independent functional localizer, see Methods) per region of interest, but the effects were largely independent of the number of voxels included. Irrespective of exact voxel selection criteria, the expectation effect (activity for non-predictable—predictable blocks) was different from zero for the perceptual task, but overlapping with zero for the working memory and grating tasks (see Fig 2B).

In addition to the interaction between task and expectation, task itself had a strong effect on the BOLD response of voxels in the primary visual cortex (main effect of task, rmANOVA over all tasks, V1: *F*_2,64_ = 21.51, *p* < .001; V2: *F*_2,64_ = 52.80, *p* < .001; V3: *F*_2,64_ = 60.47; *p* < .001; see Fig 2A). Neural activity within grating-responsive voxels in early visual cortex was more than twice as high during the grating task (i.e., when the grating stimulus was task-relevant) than during the fixation tasks (when the grating was a task-irrelevant background stimulus: *t*_32_ = 7.94, *p* < 0.0001), indicating that this task successfully manipulated spatial attention. Additionally, in V2, the comparison between fixation tasks revealed a trend of overall stronger suppression of the grating during the perceptual load task than during the working memory load task (main effect of task, rmANOVA over fixation tasks: *F*_1,32_ = 3.23, *p* = 0.082). This trend was not present in V1 (*F*_1,32_ = 0.41, *p* = 0.53) or V3 (*F*_1,32_ = 1.36, *p* = 0.25). Because tasks were manipulated between runs, the absence of a robust task effect could therefore be caused by the increased variance in the task comparisons.

#### Whole-brain activity

**Fig 3:**
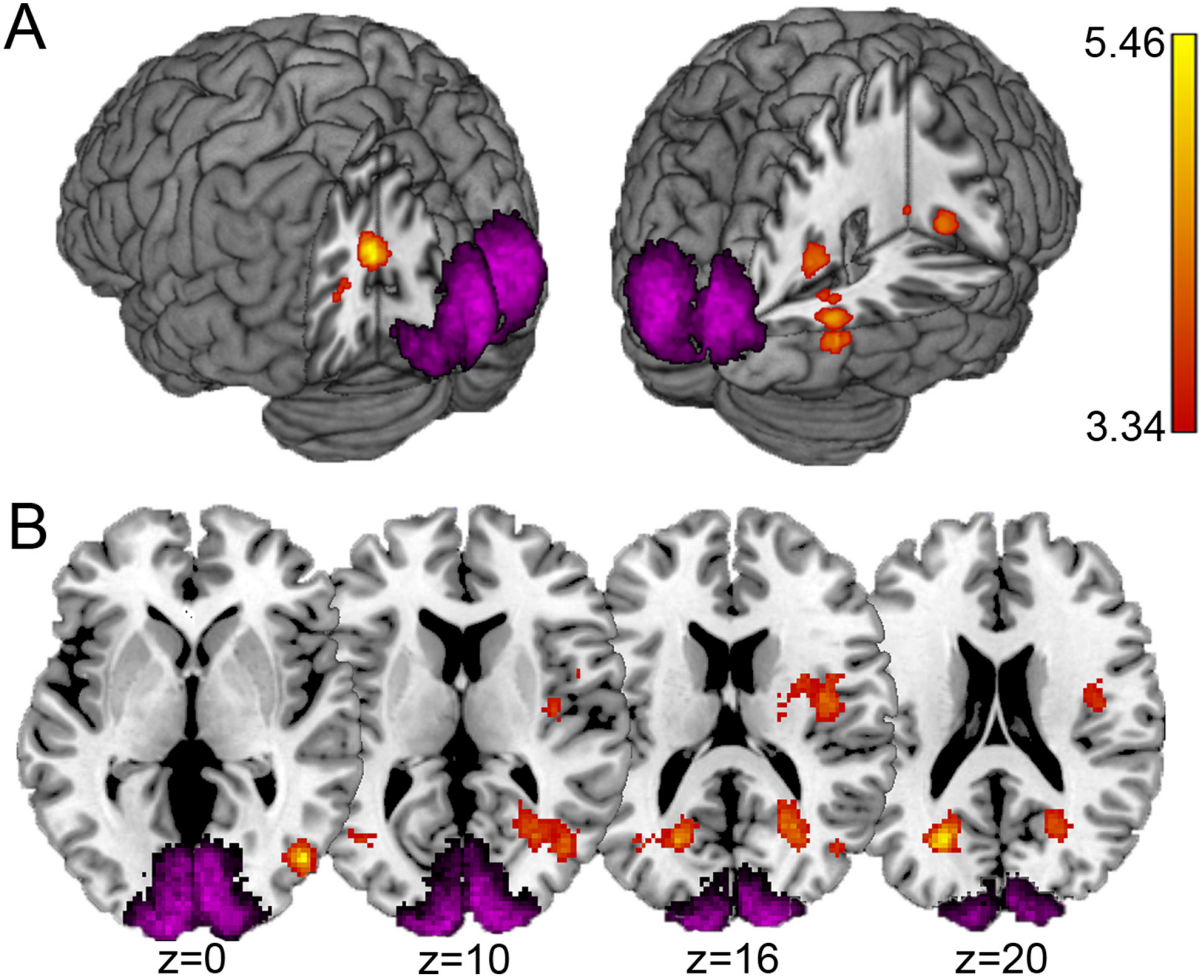
Interaction between task and expectation within bilateral cuneus and right insula. Clusters within bilateral cuneus and right insular cortex show significantly stronger expectation suppression during the perceptual task than the working memory task (red/yellow). Cuneus activity is anterior and lateral to early visual cortex (anatomical contours in magenta; saturation indicates degree of overlap between subjects). (A) Results displayed on a 3D MNI brain. (B) The same results displayed on axial slices showing the bilateral cuneus clusters and right insular cortex activation. Z-coordinates displayed under each slice in mm. Cluster extents determined by *p* < .001 uncorrected. Scale indicates *t* values and applies to both panels.

Next, we probed whether other brain regions showed a similar interaction between the relative load on perceptual and working memory resources and expectation suppression. This interaction was present in bilateral cuneus (right: *p*_*FWE*_ < .001; left: *p*_*FWE*_ = 0.013) as well as right insular cortex (*p*_*FWE*_ = 0.014, Fig 3). In other words, bilateral occipital cortex and right insula showed stronger expectation suppression in the perceptual than in the working memory task, similar to what we observed in V1-V3.

## Discussion

Expected stimuli often evoke a reduced sensory response compared to unexpected stimuli. Here we investigated whether the expectation suppression of unattended and irrelevant stimuli in the periphery depends on the type of task people perform centrally. Specifically, we compared the neural response evoked by predictable and non-predictable stimuli during tasks that loaded either perceptual resources or working memory resources. We observed that expectation significantly suppressed peripheral stimuli only during the perceptual task, in which perceptual resources were heavily loaded and working memory resources were largely available. On the other hand, when working memory resources were heavily taxed and perceptual resources were largely available, expectation suppression was abolished.

What could be the functional role of sensory suppression? Previous studies have found that sensory expectations facilitate perceptual inference, especially when input is complex and noisy or ambiguous [24]. Anticipating sensory input naturally allocates processing resources in an efficient manner, with expected stimuli (that are thus low in informative value) receiving relatively fewer resources than surprising (and thus more informative) inputs [3–5, 7, 8, 25–30]. Predicting sensory input consequently minimizes both sensory uncertainty and processing resources consumed. For these reasons, it appears advantageous for the system to suppress predictable sensory inputs whenever possible.

During the task that placed a high load on working memory resources, there was no evidence of expectation suppression of the peripheral stimuli. Why would expectation suppression be abolished when working memory resources are strongly loaded by a concurrent task? The answer may lie in the fact that both expectation and working memory rely on a common process: bringing online stimulus templates in visual cortex. Recent studies show that the working memory maintenance of visual items leads to a cortical reinstatement of the maintained material in early sensory areas [31–35], suggesting that the same neurons driven by bottom-up stimulus input become active during recall. Interestingly, stimulus expectation may employ this same process of cortical reinstatement [36, 37], with early sensory neurons representing a template of the expected stimulus, in the absence of bottom-up input [14]. Once online, such stimulus templates can then be compared with incoming sensory information, facilitating perception and behaviour when there is a match [3, 24, 29, 30, 38]. We therefore speculate that expectation suppression may be abolished during the working memory task because this task places a high demand on the same resources needed for cortical reinstatement of expectation templates of the peripheral, irrelevant stimuli. Consequently, this inability to bring online templates of expected stimuli abolishes expectation suppression. Given that the task-relevant stimuli and the gratings were processed in non-overlapping regions of V1, the conflict may not be at the level of sensory cortex, but rather at the level of the frontal and parietal regions that are involved both in working memory [39, 40] and the generation of sensory predictions [41, 42]. The prefrontal cortex is known to be involved in top-down driven distractor suppression [43, 44], and taxing this region with a working memory task may thereby hinder effective top-down suppression. Supporting this link between working memory and top-down suppression mechanisms, it has been found that during a challenging cognitive control task, individuals with high working memory capacity display relatively more distractor suppression than individuals with low working memory capacity [45].

Alternatively, the lack of expectation suppression during the working memory task may not solely be due to the high load on working memory resources, but caused by the relative absence of load on perceptual resources posed by this task. In other words, expectation suppression may take place only when predictable stimuli compete for task-relevant perceptual resources, as is the case in our perceptual load condition. During the (perceptually simple) working memory task, perceptual resources are untaxed; therefore there is no need to suppress the irrelevant peripheral grating stimuli. In line with this, there was a trend towards an overall reduced sensory response to the peripheral grating when perceptual resources were centrally loaded (i.e., the grating evoked the least neural activity while participants performed the perceptual task compared to the other two tasks). This explanation could also explain the lack of expectation suppression during the grating task, since in this case the predictable stimuli are task-relevant, and so do not induce perceptual competition. However, previous research has shown that expectation suppression is also present in the absence of perceptual competition [3, 4, 8]. We cannot distinguish between the working memory and the perceptual competition explanations of our results on the basis of the current study.

This study manipulated cognitive load type by including different types of tasks and matching them for overall difficulty using an adaptive staircase procedure. Future studies may examine the role of different types of cognitive load more fully by manipulating the level of load within tasks (i.e., high vs. low load for each task). This may allow for more specific conclusions to be drawn about the factors relevant to expectation suppression, and distinguish between the two explanations of our results presented above.

Our results suggest that expectation suppression does not require attention to the predictable stimuli, but instead depends on the type of available cognitive resources. However, one might argue that the working memory task was more demanding than the perceptual task, and therefore left less attention for the gratings. In other words, the gratings might have been somewhat attended during the perceptual task, but not during the working memory task, culminating in expectation suppression for the former task but not the latter. In contrast to this notion however, we find an overall relatively *stronger* response to the irrelevant gratings during the working memory task, opposite to what would be expected with increased attentional load at the fovea. Additionally, accuracy was matched between tasks, suggesting that the complexity of the tasks were not markedly different. Both of these points argue against the possibility of reduced spatial attention for the grating during the working memory task.

Whole brain analyses revealed that bilateral cuneus and right insula showed the same interaction pattern between expectation and task that was present in early visual cortex. These areas have been previously reported to show sensitivity to statistical regularities in the environment [46].

We also included a task where participants responded to the grating stimuli, causing the predictable stimuli to become attended. Here we did not find an effect of expectation on the neural response. This result is perhaps unexpected, given that several previous studies show expectation suppression also for attended stimuli [3–5]. However, the interaction between attention and expectation is complex and multifaceted [27, 36]. Recent studies suggest that attention interacts with expectation and may even reverse its effect [25, 47], such that expectation enhances the response when stimuli are attended [48, 49]. Indeed, many studies on attention employ cues that concurrently signal what is likely and what is relevant [50], thereby conflating expectation and attention. These studies generally observe increased activity for expected/attended sensory events [51]. In light of this, we speculate that the absence of an expectation effect for attended stimuli in the current study could potentially be explained by an interaction between the opposing effects of attention and expectation. Additionally, previous research has generally focused on the contrast between confirmed and violated expectations (i.e., expected vs. *un*expected), whereas here we contrasted situations with and without expectation cues (i.e., expected vs. *non*-expected). It is possible that the expectation suppression is particularly visible when contrasted against stimuli for which the expectation is violated, rather than for stimuli for which no strong expectation is present.

To summarize, our results provide insight into the resource dependency of expectation suppression. We show that expectation suppresses unattended, predictable stimuli when perceptual resources are loaded, but that this suppression is absent when working memory resources are loaded. This suggests that differences in the type of cognitive load might explain the conflict in the literature regarding the presence or absence of expectation suppression. Furthermore, our findings indicate that predictable stimuli do not need to be attended in order to be suppressed by expectation, pointing to resource availability as a crucial factor instead.
